# Covalent Organic Frameworks—Organic Chemistry Beyond the Molecule

**DOI:** 10.3390/molecules22091575

**Published:** 2017-09-19

**Authors:** Christian S. Diercks, Markus J. Kalmutzki, Omar M. Yaghi

**Affiliations:** 1 Department of Chemistry, University of California, Berkeley, CA 94720, USA; mjkalmutzki@berkeley.edu; 2 Materials Sciences Division, Lawrence Berkeley National Laboratory, Berkeley, CA 94720, USA; 3 Kavli Energy NanoSciences Institute, University of California, Berkeley, CA 94720, USA

**Keywords:** reticular chemistry, covalent organic frameworks, organic chemistry

## Abstract

The synthesis of organic molecules has at its core, purity, definitiveness of structure, and the ability to access specific atoms through chemical reactions. When considering extended organic structures, covalent organic frameworks (COFs) stand out as a true extension of molecular organic chemistry to the solid state, because these three fundamental attributes of molecular organic chemistry are preserved. The fact that COFs are porous provides confined space within which molecules can be further modified and controlled.

## 1. Introduction

Throughout the 20th century, the large toolbox of covalent organic transformations and the precision with which molecules can be synthesized or functionalized has matured to the point of rational synthesis [1,2]. Up until recently, this degree of design of organic matter has been limited to discrete compounds and polymers. As pointed out by Roald Hoffmann: “Organic chemists are masterful at exercising control in zero dimensions. One subculture of organic chemists has learned to exercise control in one dimension. These are polymer chemists, the chain builders. … But in two or three dimensions, it’s a synthetic wasteland” [3]. In order to devise strategies to propel organic chemistry into the realm of extended structures, the question becomes what are the underlying principles that define and represent the power of organic chemistry. In our opinion, three main aspects need to be preserved when expanding the fundamental principles of this discipline to extended structures: (i) The targeted synthesis of structures with atomic precision; (ii) definitiveness of structure to relate structure to function; and (iii) accessibility of atoms within organic structures for further chemical modification without losing control over their connectivity. We believe covalent organic frameworks (COFs) are a class of materials fulfilling these criteria. They are made by reticular synthesis, where molecular building blocks are stitched together through strong covalent bonds into frameworks of a specific targeted structure [4]. The fact that they are obtained in crystalline form complies with the requirement of structural definitiveness. Last, but not least, since these frameworks are porous and have architectural stability, that translates into the pores being accessible to incoming guests, which is necessary for consecutive post-synthetic modification. In this contribution, we put forward the idea that COFs are the only class of organic materials that affords extended structures which preserve all three of the aforementioned criteria and give illustrative examples of how this approach has been applied to the formation of three-dimensional (3D) [5], two-dimensional (2D) [6], and one-dimensional (1D) [7] extended structures.

## 2. Definitiveness of Structure—Crystallinity

**Figure 1:**
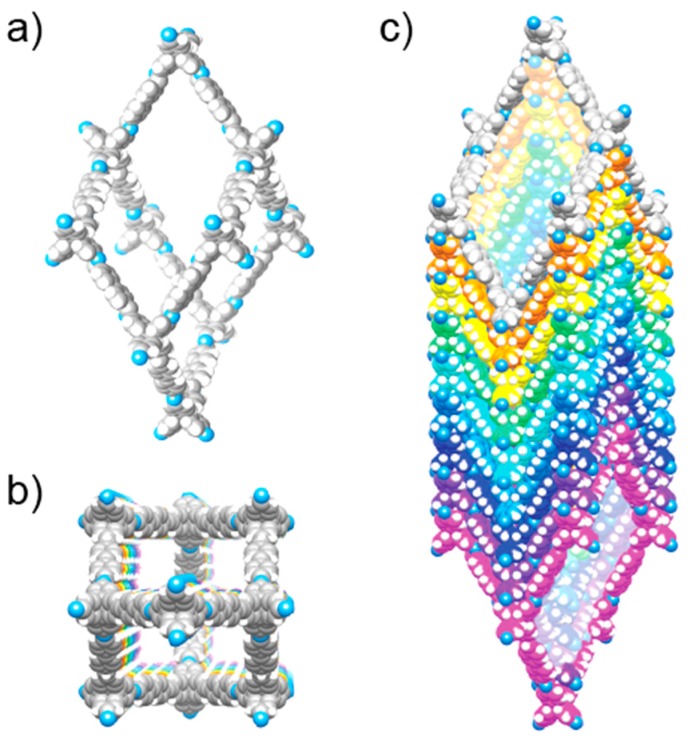
Crystal structure of COF-320 obtained by rotating electron diffraction (RED) at 298 K. (**a**) Adamantane-cage of the diamond net; (**b**) Representation of the nine-fold interpenetration of the diamond net, viewed along the crystallographic *c*-direction; (**c**) The square-shaped, one-dimensional (1D) channels running along the *c*-axis.

In essence, molecules are geometric constructs of atoms. The precise arrangement of these atoms is essential to the extent that the exact constitution or even their stereochemistry can determine whether a molecule is a poison or a drug [8]. To correlate structure to function, one needs to obtain and characterize phase-pure products. Hence, the synthesis of organic molecules is generally followed by purification (e.g., column chromatography, recrystallization, etc.) and subsequent characterization of the pure product. This is why the rapid development of organic synthetic methodologies throughout the 20th century went hand in hand with important developments in spectroscopic characterization techniques. In particular, the use of nuclear magnetic resonance spectroscopy gave a handle on characterizing the connectivity within discrete organic compounds to deduce their structure. In the case of extended solids, however, various structure types with identical composition can be formed. For example, the linking of tetrahedral molecules may result in as many as several million different structure types. Here, the connectivity is not enough to unambiguously determine the structure and, for extended frameworks, crystallinity is therefore a necessary requirement for structure elucidation. In addition, crystallinity assures the long-range order and uniformity of the molecular arrangement, which is crucial for structure-property correlations. Extended structures are generally insoluble. As such, the product cannot be recrystallized and the reticulation step and the crystallization necessarily need to occur simultaneously. The importance of crystallinity in elucidating the structure of 3D extended organic frameworks can be illustrated with the example of COF-320, a material synthesized by the condensation of tetra-(4-anilyl)methane and 4,4′-biphenyldialdehyde into an imine-linked three-dimensional extended structure, based on a diamond net [9]. Using rotating electron diffraction (RED) at 298 K, the crystal structure of COF-320 could unambiguously be determined. The dimensions of the individual adamantane cages in the framework are 28 × 31 × 71 Å^3^ and, due to the large void space within these cages, the structure is isolated as a nine-fold interpenetrated framework (Figure 1). In this example, the high degree of interpenetration precludes an unambiguous structure solution without crystallographic data. The crystallization of extended organic materials is challenging, and the linking of molecular building blocks by strong covalent bonds often yields amorphous or poorly defined materials such as microporous polymers [10]. As illustrated above, these materials lose the definitiveness of structure which is at the very core of synthetic organic chemistry and as such, by our definition, do not constitute a true extension of organic chemistry to the solid state.

## 3. Targeted Covalent Synthesis

**Figure 2:**
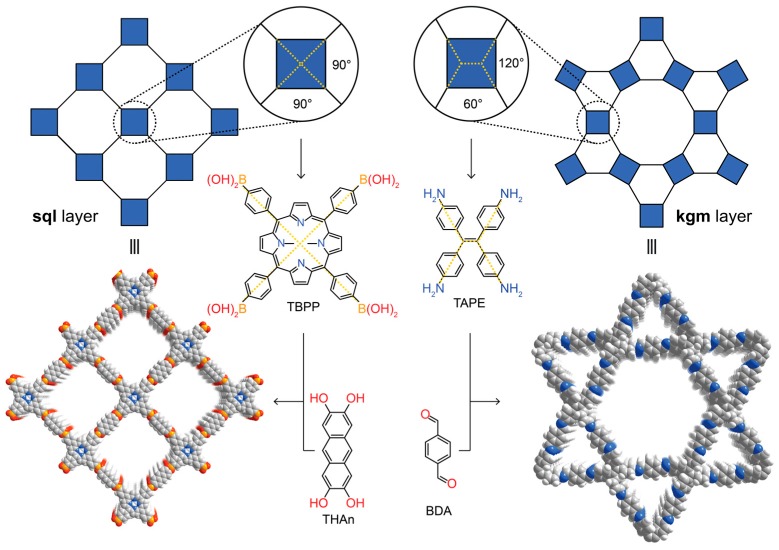
Targeted reticular retrosynthesis of covalent organic frameworks with different topologies but with the same connectivity. Precise adjustment of the angles in the starting materials yields covalent organic frameworks (COFs) of square lattice (COF-66) or kagome topology (4PE-1P-COF).

The targeted synthesis of organic compounds relies on utilizing the high directionality of covalent bonds to allow for the prediction of the geometric arrangement of the atoms in the product. The advent of supramolecular chemistry—the chemistry beyond the molecule—was widely expected to bring about synthetic strategies to expand molecular organic chemistry to the solid state [11,12,13,14]. However, while many extended supramolecular structures can be crystallized, the low directionality of the weak, non-covalent interactions inherent to this approach are often at the mercy of randomness and fail to generate generalizable synthetic protocols since modification of the building blocks inevitably alters the interactions between the constituents, which in turn leads to different molecular assemblies. In COFs, owing to the directionality of covalent bonds, the connectivity and geometry of the building blocks can be chosen systematically to target specific structure types. In essence, just like molecules are geometric constructs of atoms, COFs can be considered geometric constructs of molecules. The power of the covalent bond in this reticulation process becomes evident when targeting frameworks of a specific structure type. For example, forming 2D layered structures using tetratopic building blocks can theoretically lead to two different topologies—square lattice (**sql**) or kagome (**kgm**) layers. In the chemistry of COFs, basic geometric considerations facilitate targeting one structure type over the other. This is illustrated with the example of COF-66 and 4PE-1P-COF. In the highest symmetry embedding of the **sql** net, the angles between the points of extension of the tetratopic building block have to be 90°, which is found in porphyrin derivatives. Consequently, reticulating tetra-(4-boronic-acid-phenyl)porphyrin with the linear 2,3,4,5-tetrahydroxyanthracene leads to the **sql** topology COF-66 [6]. In contrast, a **kgm** layer has alternating 120° and 60° angles between the points of extension of the tetratopic building block. These angles can be found in tetra-(4-aminophenyl)ethylene and indeed, reticulation of this building block with terephthaldehyde results in the formation of 4PE-1P-COF with **kgm** topology (Figure 2) [15,16]. This approach is considered as the extension of retrosynthesis from molecules to extended structures. More recently, alternative strategies of forming crystalline 2D polymers which rely on separating the crystallization from the polymerization step have been reported. Here, molecules are crystallized in a specific spatial arrangement by careful modulation of the intermolecular interactions between the constituents (and the solvent of crystallization), and only then can the resulting molecular crystal be polymerized in a single crystal to single crystal transformation using photodimerization reactions [17,18]. It must be noted, however, that this approach relies on non-covalent bonds with limited directionality in the crystallization process. This severely limits the predictability of the outcome of the initial crystallization step. While this approach may occasionally yield highly crystalline materials, it is impossible to dial in subtle differences such as the required angles for the different topologies mentioned above, which prevents the rational design of targeted structure types and thus a true retrosynthetic approach to the design of 2D polymers.

## 4. Accessibility of the Constituents through the Use of Porosity

**Figure 3:**
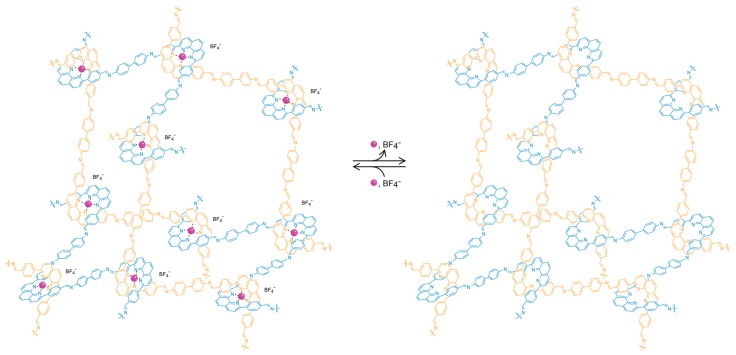
Illustration of the adamantane cages of metalated and unmetalated COF-505. The open structure of the framework allows for reversible post-synthetic metalation/demetalation with full retention of the overall structure.

Finally, the last criterion that we proposed above is the necessity for accessibility of the constituents of the extended solid to allow for further chemical transformations. In molecular chemistry, this is achieved by carrying out reactions in solution which enable the access of reagents to the substrate. In extended structures, this task is more challenging, particularly for 2D and 3D extended structures, as they tend to be insoluble. In COFs, this accessibility is provided by the porous nature of the frameworks, leading to the facile diffusion of reagents throughout the entire material. Indeed, the porosity serves an additional purpose as it provides for the necessary space to accommodate for the appended functionality without changing the metrics of the overall structure [19,20,21]. This is an important point to consider because rational optimization of a given framework becomes possible as opposed to the formation of an entirely different structure. Here, it is worthwhile to consider the case of crystalline 1D polymers, which are oftentimes credited as the extension of organic chemistry to extended structures due to the undeniable definitiveness of their structure. However, these 1D structures tend to prefer to pack in a dense, parallel arrangement, preventing their functionalization in the solid state. In an attempt to expand the chemistry of COFs to 1D extended structures, COF-505, a framework constructed from the weaving of organic threads, was synthesized by the imine condensation of aldehyde functionalized copper(I)-bisphenanthroline tetrafluoroborate with benzidine, resulting in a one-fold interpenetrated structure of diamond topology (Figure 3) [7]. The copper centers serve as templates for bringing the threads into a woven pattern, as opposed to the more commonly observed parallel arrangement, and can be reversibly removed and added without deterioration of the overall COF structure. That the removal of the copper centers is fully reversible is credited to the fact that the global arrangement of the 1D polymer chains in the solid remains unchanged throughout the metalation/demetalation process. In addition, the notion that this reaction can be carried out on a solid material shows the importance of open framework structures in the context of post-synthetic modification. To the best of our knowledge, there is no generalizable approach to control the packing of polymer chains on the molecular level, and COF-505 is therefore a compelling illustration of the power of reticular synthesis with regard to the design of new extended organic materials based on 1D objects.

## 5. Conclusions

The reticular synthesis of COFs allows for the targeted synthesis of extended 3D, 2D, and 1D structures from molecular building blocks. We propose that these materials are the only true extension of synthetic organic chemistry to the solid state for the three reasons outlined above. The fact that COFs are crystalline ensures the unambiguous confirmation of their structure. In addition, the fact that their backbones are entirely composed of directional covalent bonds gives a handle on the design of the spatial orientation of the building blocks in respect to each other by rationally targeting specific structure types. Finally, since COFs are made from polyatomic building blocks, their structures feature open channels or cages for the facile diffusion of reagents to take place, thus preserving the prospect of consecutive organic functionalization of the backbone of structures without changing their underlying topology or metrics. It is noteworthy that COFs should not merely be considered an expansion of organic chemistry from discrete molecules to extended structures, but that indeed this field surpasses the level of synthetic control of molecular organic chemistry in that, in COFs, not only matter but also the encompassed empty space can be controlled with unprecedented precision, and can be further used to manipulate matter on the molecular level.

## References

[1] Corey E.J. (1989). The Logic of Chemical Synthesis.

[2] Woodward R.B. (1973). The total synthesis of vitamin B12. Pure Appl. Chem..

[3] Hoffmann R. (1993). How should chemists think?. Sci. Am..

[4] Diercks C.S., Yaghi O.M. (2017). The atom, the molecule, and the covalent organic framework. Science.

[5] El-Kaderi H.M., Hunt J.R., Mendoza-Cortés J.L., Côté A.P., Taylor R.E., O’Keeffe M., Yaghi O.M. (2007). Designed synthesis of 3D covalent organic frameworks. Science.

[6] Cote A.P., Benin A.I., Ockwig N.W., O’Keeffe M., Matzger A.J., Yaghi O.M. (2005). Porous, crystalline, covalent organic frameworks. Science.

[7] Liu Y., Ma Y., Zhao Y., Sun X., Gándara F., Furukawa H., Liu Z., Zhu H., Zhu C., Suenaga K. (2016). Weaving of organic threads into a crystalline covalent organic framework. Science.

[8] Maio G. (2001). On the history of the Contergan (thalidomide) catastrophe in the light of drug legislation. Dtsch. Med. Wochenschr..

[9] Zhang Y.-B., Su J., Furukawa H., Yun Y., Gándara F., Duong A., Zou X., Yaghi O.M. (2013). Single-crystal structure of a covalent organic framework. J. Am. Chem. Soc..

[10] Wu D., Xu F., Sun B., Fu R., He H., Matyjaszewski K. (2012). Design and preparation of porous polymers. Chem. Rev..

[11] Pedersen C.J. (1967). Cyclic polyethers and their complexes with metal salts. J. Am. Chem. Soc..

[12] Dietrich B., Lehn J., Sauvage J., Blanzat J. (1973). Cryptates—X: Syntheses et proprietes physiques de systemes diaza-polyoxa-macrobicycliques. Tetrahedron.

[13] Cram D.J. (1988). The design of molecular hosts, guests, and their complexes (Nobel lecture). Angew. Chem. Int. Ed. Engl..

[14] Kinoshita Y., Matsubara I., Higuchi T., Saito Y. (1959). The crystal structure of bis (adiponitrilo) copper (I) nitrate. Bull. Chem. Soc. Jpn..

[15] Zhou T.-Y., Xu S.-Q., Wen Q., Pang Z.-F., Zhao X. (2014). One-step construction of two different kinds of pores in a 2D covalent organic framework. J. Am. Chem. Soc..

[16] Ascherl L., Sick T., Margraf J.T., Lapidus S.H., Calik M., Hettstedt C., Karaghiosoff K., Döblinger M., Clark T., Chapman K.W. (2016). Molecular docking sites designed for the generation of highly crystalline covalent organic frameworks. Nat. Chem..

[17] Kissel P., Murray D.J., Wulftange W.J., Catalano V.J., King B.T. (2014). A nanoporous two-dimensional polymer by single-crystal-to-single-crystal photopolymerization. Nat. Chem..

[18] Kissel P., Erni R., Schweizer W.B., Rossell M.D., King B.T., Bauer T., Götzinger S., Schlüter A.D., Sakamoto J. (2012). A two-dimensional polymer prepared by organic synthesis. Nat. Chem..

[19] Waller P.J., Lyle S.J., Osborn Popp T.M., Diercks C.S., Reimer J.A., Yaghi O.M. (2016). Chemical Conversion of Linkages in Covalent Organic Frameworks. J. Am. Chem. Soc..

[20] Huang N., Krishna R., Jiang D. (2015). Tailor-made pore surface engineering in covalent organic frameworks: Systematic functionalization for performance screening. J. Am. Chem. Soc..

[21] Ding S.-Y., Gao J., Wang Q., Zhang Y., Song W.-G., Su C.-Y., Wang W. (2011). Construction of covalent organic framework for catalysis: Pd/COF-LZU1 in Suzuki–Miyaura coupling reaction. J. Am. Chem. Soc..

